# The Antioxidant, MnTE-2-PyP, Prevents Side-Effects Incurred by Prostate Cancer Irradiation

**DOI:** 10.1371/journal.pone.0044178

**Published:** 2012-09-12

**Authors:** Rebecca E. Oberley-Deegan, Joshua J. Steffan, Kyle O. Rove, Kathryn M. Pate, Michael W. Weaver, Ivan Spasojevic, Barbara Frederick, David Raben, Randall B. Meacham, James D. Crapo, Hari K. Koul

**Affiliations:** 1 Pulmonary Division, Department of Medicine, National Jewish Health, Denver, Colorado, United States of America; 2 Program in Urosciences, Division of Urology, Department of Surgery, School of Medicine, University of Colorado Anschutz Medical Campus, Aurora, Colorado, United States of America; 3 Denver Veterans Administration Medical Center, Denver, Colorado, United States of America; 4 Department of Medicine, Duke University Medical Center, Durham, North Carolina, United States of America; 5 PD/PD Bioanalytical Core Laboratory, Duke Cancer Institute, Durham, North Carolina, United States of America; 6 Radiation and Cancer Biology Division, Department of Cancer Biology, University of Colorado Health Sciences Center, Aurora, Colorado, United States of America; 7 University of Colorado Comprehensive Cancer Center, Aurora, Colorado, United States of America; Florida International University, United States of America

## Abstract

Prostate cancer is the most commonly diagnosed cancer, with an estimated 240,000 new cases reported annually in the United States. Due to early detection and advances in therapies, more than 90% of patients will survive 10 years post diagnosis and treatment. Radiation is a treatment option often used to treat localized disease; however, while radiation is very effective at killing tumor cells, normal tissues are damaged as well. Potential side-effects due to prostate cancer-related radiation therapy include bowel inflammation, erectile dysfunction, urethral stricture, rectal bleeding and incontinence. Currently, radiation therapy for prostate cancer does not include the administration of therapeutic agents to reduce these side effects and protect normal tissues from radiation-induced damage. In the current study, we show that the small molecular weight antioxidant, MnTE-2-PyP, protects normal tissues from radiation-induced damage in the lower abdomen in rats. Specifically, MnTE-2-PyP protected skin, prostate, and testes from radiation-induced damage. MnTE-2-PyP also protected from erectile dysfunction, a persistent problem regardless of the type of radiation techniques used because the penile neurovascular bundles lay in the peripheral zones of the prostate, where most prostate cancers reside. Based on previous studies showing that MnTE-2-PyP, in combination with radiation, further reduces subcutaneous tumor growth, we believe that MnTE-2-PyP represents an excellent radioprotectant in combination radiotherapy for cancer in general and specifically for prostate cancer.

## Introduction

Prostate cancer is the second most common type of cancer for men in Western countries [1]. Radiation therapy is routinely used to treat prostate cancer in men [2]. Although radiation effectively kills prostate tumor cells, it also inadvertently damages surrounding tissues. Long-term complications from radiation therapy directed to the prostate region are bowel and rectal wall damage, lower urinary tract symptoms such as urgency and frequency, erectile dysfunction, urethral stricture, and incontinence [3], [4]. This damage can occur anytime during radiation therapy and has been reported years after treatment [5]. Once the damage of normal tissue begins, it is usually progressive and irreversible. A persistent problem after high dose radiation is progressive erectile dysfunction. In fact, a recent report using the PROSTQA (Prostate Cancer Outcomes and Satisfaction with Treatment Quality Assessment) cohort of patients has shown that 63% of patients receiving external radiotherapy and 57% of patients receiving brachytherapy reported erectile dysfunction two years after treatment. The reason lies in the fact that neurovascular bundles which are involved in the erectile process lie primarily in the peripheral zones where most prostate cancers reside. Because people are living longer after treatment of cancer, radiation therapy-induced quality of life impairments are becoming increasingly important to address.

Although the exact mechanisms underlying radiation-induced injury of the healthy, surrounding tissue are not known, many studies implicate free radicals as a cause of radiation-induced tissue damage [6]. After irradiation of normal tissues, an acute inflammatory response is followed by a chronic inflammatory/wound healing response. The irreversible tissue damage associated with radiation is caused by the chronic inflammatory response [7]. A number of studies have implicated oxidative stress as driving both the acute and chronic inflammation associated with radiation-induced tissue damage [7]. Specifically, Kimura *et al.* have recently shown reactive oxygen species (ROS) as a major driver of erectile dysfunction induced by irradiation [8], [9]. Thus, the use of antioxidants, molecules that remove free radicals, in combination with radiation therapy should minimize radiation-induced injury to normal tissues.

MnTE-2-PyP (chemical name: Manganese (III) *Meso*-Tetrakis-(N-Methylpyridinium-2-yl) porphyrin) is a potent, small molecular weight antioxidant that scavenges a variety of free radicals including superoxide, hydrogen peroxide, lipid peroxides and peroxynitrite [10], [11], [12], [13], [14]. MnTE-2-PyP reduces inflammation and injury in a variety of models including bleomycin and lipopolysaccharide exposure [15], [16]. One way MnTE-2-PyP reduces inflammation is by inhibiting NF-κB signaling by altering the redox environment around the transcription factor [16]. It is believed that MnTE-2-PyP is able to affect such a wide variety of different disease states through its ability to alter cell signaling pathways. Its chemistry, mechanism of action, bioavailability and therapeutic applications, as well as its Mn porphyrin analogs have been recently reviewed [17], [18].

Recently, MnTE-2-PyP, has been shown to prevent radiation-induced tissue injury. The antioxidant prevents damage to photoreceptors and retinal capillaries from eye irradiation and similarly prevents radiation-induced damage to the lung [19], [20]. However, in order for a drug to be used as a potential radioprotectant in cancer patients, it is imperative that the drug does not protect the tumor cells from injury caused by irradiation. Gridley *et al.* have shown that prostate tumors in mice treated with a combination of radiation and MnTE-2-PyP, grew more slowly than tumors treated with radiation alone [21]. Therefore, these previous data suggest that MnTE-2-PyP protects normal tissues from radiation-induced damage, but does not protect tumor cells from radiation-induced death.

We hypothesized that MnTE-2-PyP would protect the urogenital system from damage associated with pelvic irradiation. To test this hypothesis, we exposed rats to fractionated irradiation of the pelvic region with and without MnTE-2-PyP administration. We harvested animals 12 weeks post-irradiation and found that MnTE-2-PyP protected the structure and function of organs exposed to radiation. Specifically, MnTE-2-PyP protected the skin, prostate, testes, and penile tissues from irradiation-induced damage and prevented the loss of erectile function caused by radiation therapy. We speculate that MnTE-2-PyP could be a powerful radioprotectant when administered with prostate cancer radiation treatment in humans to further minimize long term bowel and urinary injury as well as preserve erectile function.

## Materials and Methods

### Experimental animals

Sprague-Dawley rats (100–150 g, 4–6 weeks in age) from either Jackson or Harlan Laboratories were used in this study. Rats were housed at either the University of Colorado Anschutz Medical Campus or at National Jewish Health and given a continuous supply of food and water. This study was carried out in strict accordance with the recommendations of the Guide for the Care and Use of Laboratory Animals of the National Institutes of Health. All treatments and procedures were approved by the institutional animal care and use committees at the two institutions (NJH#AS2700-05-12, UCAMC# 68810(10)1E).

### Treatment groups and experimental design

**Figure 1:**
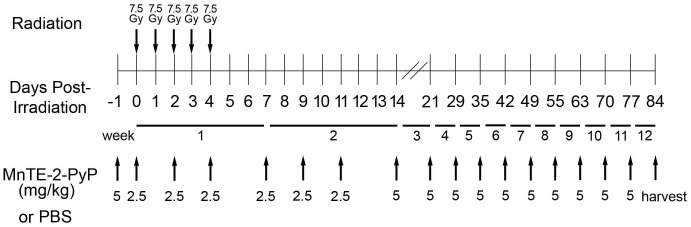
Diagram of Experimental Design. Rats received 5 consecutive radiation doses of 7.5 Gy to the lower abdomen. Rats were injected i.p. with MnTE-2-PyP or PBS as a control throughout the study as indicated. Animals were harvested 12 weeks post-irradiation. There were four animals per group and the experiment was repeated once.

Animals were randomly assigned to 4 groups (8 rats per group): 1) PBS injected with no irradiation, 2) MnTE-2-PyP injected with no irradiation, 3) PBS injected with irradiation, and 4) MnTE-2-PyP injected with irradiation. The experimental design is illustrated in Figure 1. Briefly, animals were injected with MnTE-2-PyP (5 mg/kg) or PBS 24 hours before the start of radiation. Rats were irradiated for 5 sequential days with 7.5 Gy/day in the lower pelvic region. This irradiation scheme was chosen because it mimics an irradiation scheme that a patient undergoing prostate cancer therapy would undertake to eradicate the prostate tumor [22]. MnTE-2-PyP (2.5 mg/kg) or PBS was administered every other day for the following two weeks. The animals were then administered MnTE-2-PyP (5 mg/kg) or PBS once a week until 12 weeks post-irradiation. The rats weights and radiation-induced skin damage was documented once a week. At the 12 week time point, the erectile function of the rats was accessed and tissues (prostate and penis) were harvested from the animals. This experiment was repeated once with the same groups and time points.

### MnTE-2-PyP treatment

MnTE-2-PyP was synthesized by Ricerca Biosciences, LLC. Concord, OH, USA. MnTE-2-PyP was dissolved in PBS and injected (100 µl) intraperitoneally (i.p.) at 2.5 or 5 mg/kg at the above specified times. PBS (100 µl) injected i.p. was used as a control.

### Pharmacokinetics of MnTE-2-PyP in rat urogenital system

The same MnTE-2-PyP dosing scheme was used for pharmacokinetic analysis as was used for the experimental design with a different cohort of animals. There were four groups of animals and each group had 3 rats: group 1) rats were harvested 1 week after the start of injections, group 2) rats were harvested 2 weeks after the start of injections, group 3) rats were harvested 12 weeks after the start of injections (harvested 7 days after the last injection), group 4) rats were harvested 12 weeks+one day after the start of injections (harvested 1 day after the last injection). At each time point, the liver, bowel, prostate, penile tissue and bladder were collected and flash frozen. The MnTE-2-PyP concentrations were then determined by the PK/PD Bioanalytical Core Laboratory at the Duke Cancer Institute as previously described [23].

### Scoring of skin reaction to irradiation

Animals were observed through the course of the experiment. They received a score from a reaction scale from Hall and Giaccia [24]. O = no visible reaction, 1 = faint erythema, 2 = erythema, 3 = marked erythema, 4 = moist desquamation of less than half of the irradiated area, 5 = moist desquamation of more than half the irradiated area.

### Histology of rat urogenital system

Rat tissues (prostate and penis) were fixed in 4% paraformaldehyde and embedded in paraffin. The tissue blocks were sectioned (5 µm thick), deparafinized in xylene and rehydrated through sequential steps of 100, 95, and 75% ethanol. The sections were stained with hematoxylin and eosin, dehydrated and coverslips mounted. Penile tissue was also stained with trichrome (Sigma, St. Louis, MO) to visualize fibrosis, according to the manufacturer's protocol.

### Immunohistochemistry

Immunohistochemistry was performed utilizing standard DAB techniques (Vector Labs, Burlingame, CA). Paraffin blocks were sectioned, deparafinized, and rehydrated. Antigens were unmasked with preheated antigen retrieval in sodium citrate buffer for 20 min and then cooled. H_2_O_2_ (3%) was placed on the slides for 5 minutes to block endogenous peroxides, followed by a serum block for 30 minutes. The 8-OHdG primary antibody (Abcam, Cambridge, MA, 5 µg/mL) was then added overnight at the indicated dilution. Biotinylated mouse secondary antibody (1∶200) was then added for 45 minutes. Biotin binding was increased using the ABC Elite method for 30 min and stains were visualized with DAB. The tissues were counterstained with hematoxylin, dehydrated and coversliped. The 8-OHdG stained sections were quantified by taking 10 random pictures of each prostate section. The sections were scored blindly, and any nuclear staining was counted as a positive 8-OHdG stained cell.

### Erectile functional assay

The erectile functional assay was performed in rats 12 weeks post-irradiation, as previously described [25]. Briefly, rats were anesthetized with sodium pentobarbital before dissection of the lower abdomen to locate the cavernous nerve. Two stainless steel electrodes were placed around the cavernous nerve on one side, with the negative electrode approximately 1 mm from the positive electrode. The skin overlaying the penis was removed and the crura were dissected free. A 26-guage needle that was connected to a pressure transducer and was inserted into either the right or left crus. Electrostimulation was performed using a stimulator (World Precision Instruments, Sarasota, FL, USA). Increases in intracavernous pressure were measured and recorded using the Data-Trax data acquisition software (Distributed Design Concepts, Dover, NH, USA).

## Results

**Figure 2:**
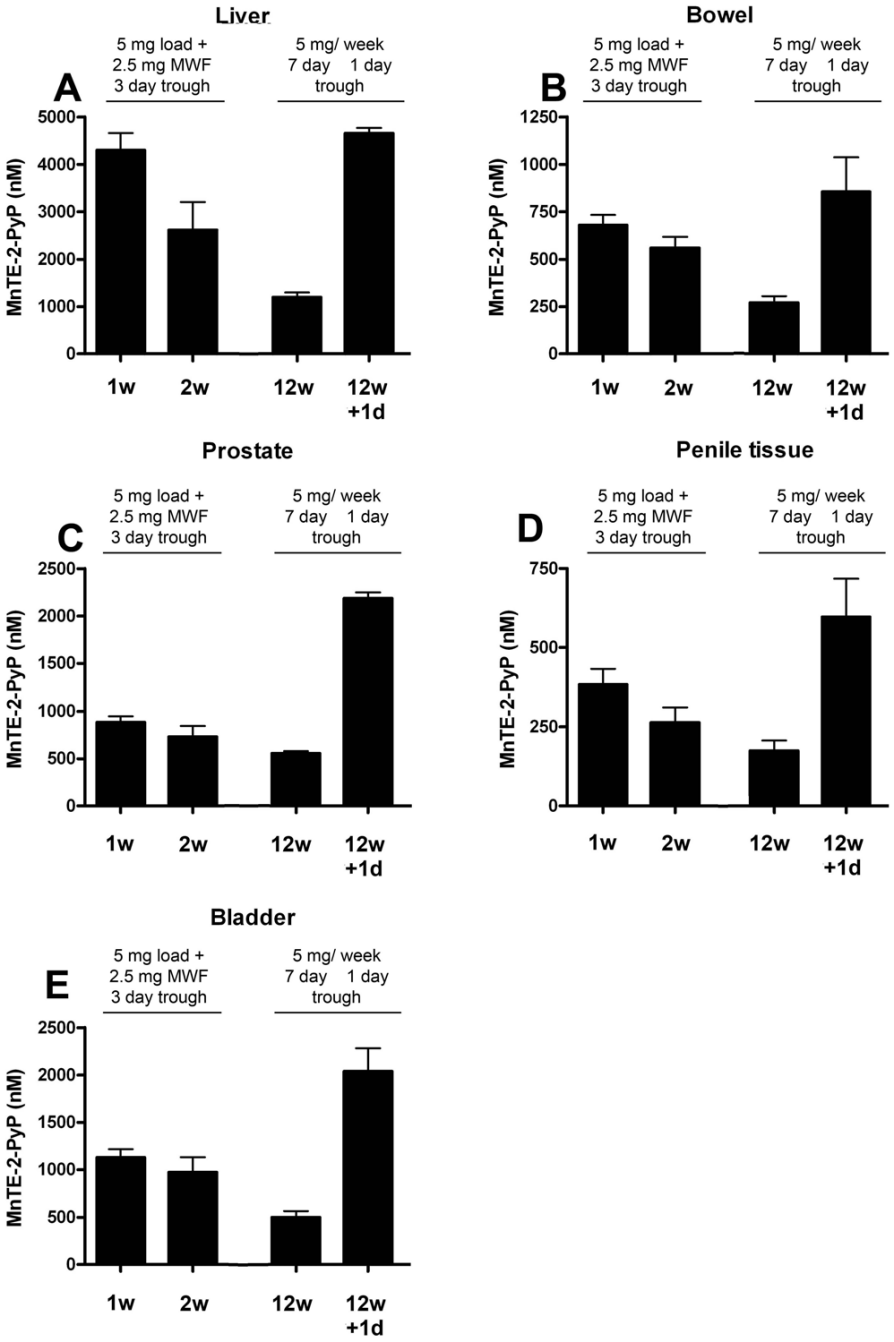
Pharmacokinetics of MnTE-2-PyP in the rat urogenital system. Rats received injections of MnTE-2-PyP as outlined in Figure 1 and harvested 1 week, 2 weeks, 12 weeks and 12 weeks+1 day after the start of injections. Rats harvested at 1 and 2 weeks had not received an injection for 3 days (3 day trough). Rats harvested at 12 weeks, had not received an injection for 7 days (representing the lowest levels during the week) and 12 weeks+1 day represent rats 1 day after an injection (representing the highest levels during the week). A. Liver. B. Bowel. C. Prostate. D. Penile tissues. E. Bladder. MnTE-2-PyP was detected in the liver, bowel, prostate, penile tissue and bladder. Data represents the mean ± standard error of the mean, n = 3 per time point.

Pharmacokinetic analysis was performed to ensure that adequate levels of MnTE-2-PyP would reach the urogenital tissues when administered intraperitoneally (i.p.). Rats were subjected to the same MnTE-2-PyP dosing scheme (Figure 1) as the animals undergoing irradiation and tissues were harvested 1 week, 2 weeks,12 weeks, and 12 weeks+one day after the start of drug administration. The liver, bowel, prostate, penis, and bladder tissues all contained MnTE-2-PyP at every time point investigated (Figure 2). The liver contained the highest concentration of MnTE-2-PyP, which is not surprising since MnTE-2-PyP is known to accumulate in the liver [23]. The bowel, prostate, and bladder all had similar levels of MnTE-2-PyP (∼1000 nM) over the course of the pharmacokinetic analysis. This concentration of MnTE-2-PyP has been shown to prevent free radical damage in other tissue types, including the lung [23], [26]. Penile tissues had the lowest levels of MnTE-2-PyP, but were still detectable. We conclude that adequate levels of MnTE-2-PyP are able to reach the urogenital system when MnTE-2-PyP is administered i.p.

**Figure 3:**
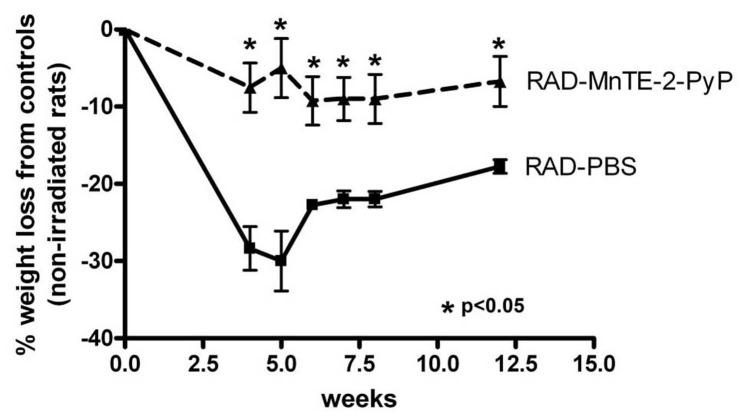
Irradiated rats receiving MnTE-2-PyP lost significantly less weight than irradiated rats injected with PBS. Weights of irradiated animals throughout the course of the experiment as compared to their respective non-irradiated groups. n = 8 rats per group, asterisk (*) denotes p<0.05.

A known side-effect of radiation exposure is weight loss. Animals were weighed throughout the course of the experiment (Figure 3). Irradiated rats lost on average 23.9% more weight than non-irradiated rats. Irradiated rats injected with MnTE-2-PyP only lost 5.9% more weight than MnTE-2-PyP injected, non-irradiated, control animals. The irradiated rats receiving MnTE-2-PyP lost significantly less weight as compared to irradiated rats not receiving MnTE-2-PyP at all time points measured (Figure 3).

**Figure 4:**
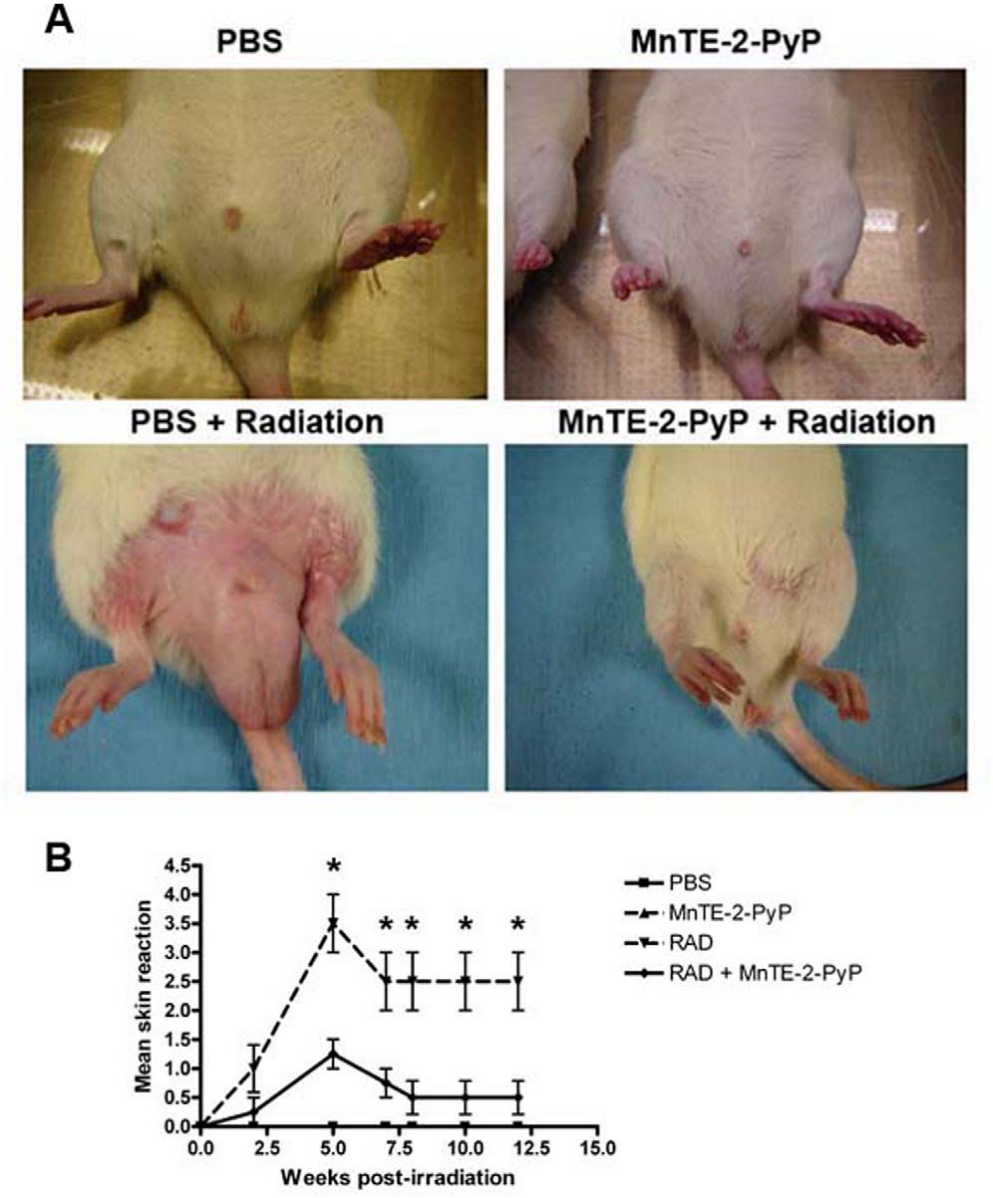
MnTE-2-PyP protected from irradiation-induced skin damage. A. Representative pictures of lower abdomens at 6 weeks post-irradiation. Irradiation caused marked epilation, which MnTE-2-PyP protected. B. Irradiation caused erythma and moist desquamation in the exposed skin exposed. The skin of irradiated rats injected with MnTE-2-PyP, had significantly less severe erythma and no moist desquamation. These skin changes persisted through the course of the experiment, n = 8 rats per group, asterisk (*) denotes p<0.05.

**Figure 5:**
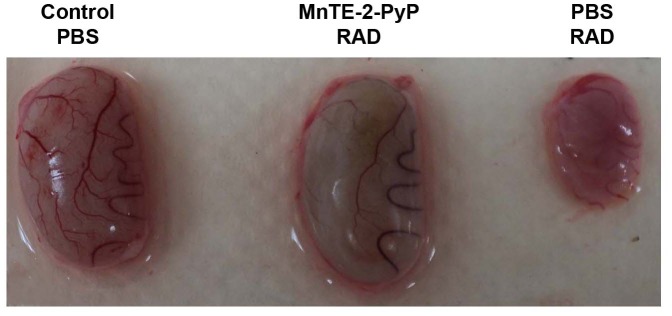
Representative pictures of rat testes at 12 weeks post-irradiation. Radiation caused the rat testes to shrink in size (∼60%, PBS RAD) as compared to non-irradiated rat testes (Control PBS). However, testes from animals treated with MnTE-2-PyP (MnTE-2-PyP RAD) were indistinguishable from non-irradiated testes.

Throughout the course of the experiment, phenotypic changes were observed between the treatment groups. Epilation was observed in the lower abdominal region in irradiated rats (Figure 4A); however, treatment with MnTE-2-PyP markedly blocked epilation in the radiation exposed area (Figure 4A). The skin of PBS injected irradiated rats had significantly more erythma and moist desquamation as compared to the skin of irradiated animals injected with MnTE-2-PyP (Figure 4B). An additional observation was a significant radiation-induced atrophy of the rat testes at 12 weeks post-irradiation (Figure 5). The testes were ∼40% the size of non-irradiated testes. However, we did not observe a change in the testis size in irradiated rats receiving MnTE-2-PyP compared to control, suggesting that MnTE-2-PyP also protects the testis from radiation-induced atrophy/damage.

**Figure 6:**
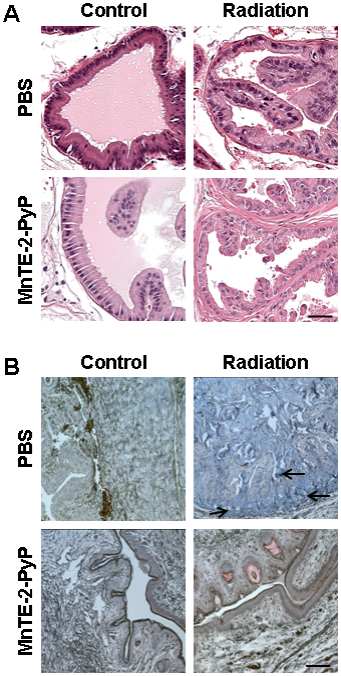
MnTE-2-PyP protects prostatic and penile tissues from irradiation-induced damage 12 weeks post-irradiation. A. Representative images of hematoxylin/eosin staining of prostate tissue (scale bar represent 5 µm). B Representative images of Masson's trichrome staining in penile tissues (scale bare represents 10 µm). The arrows indicate strong collagen staining (blue) and brown staining represents muscle fibers.

At 12 weeks post-radiation therapy, histological analysis was performed on the prostate and penile tissues. Representative images of hematoxylin and eosin staining within the prostate demonstrate that MnTE-2-PyP protected the prostate epithelial glands from atrophy and loss of prostatic epithelial architecture in the irradiated rats (Figure 6A). Moreover, penile tissues were stained using Masson's trichrome stain to characterize tissue fibrosis. The trichrome staining revealed that MnTE-2-PyP prevented radiation-induced loss of smooth muscle and accumulation of collagen in penile tissues (Figure 6B), suggesting that MnTE-2-PyP protects penile tissue from radiation-induced fibrosis.

**Figure 7:**
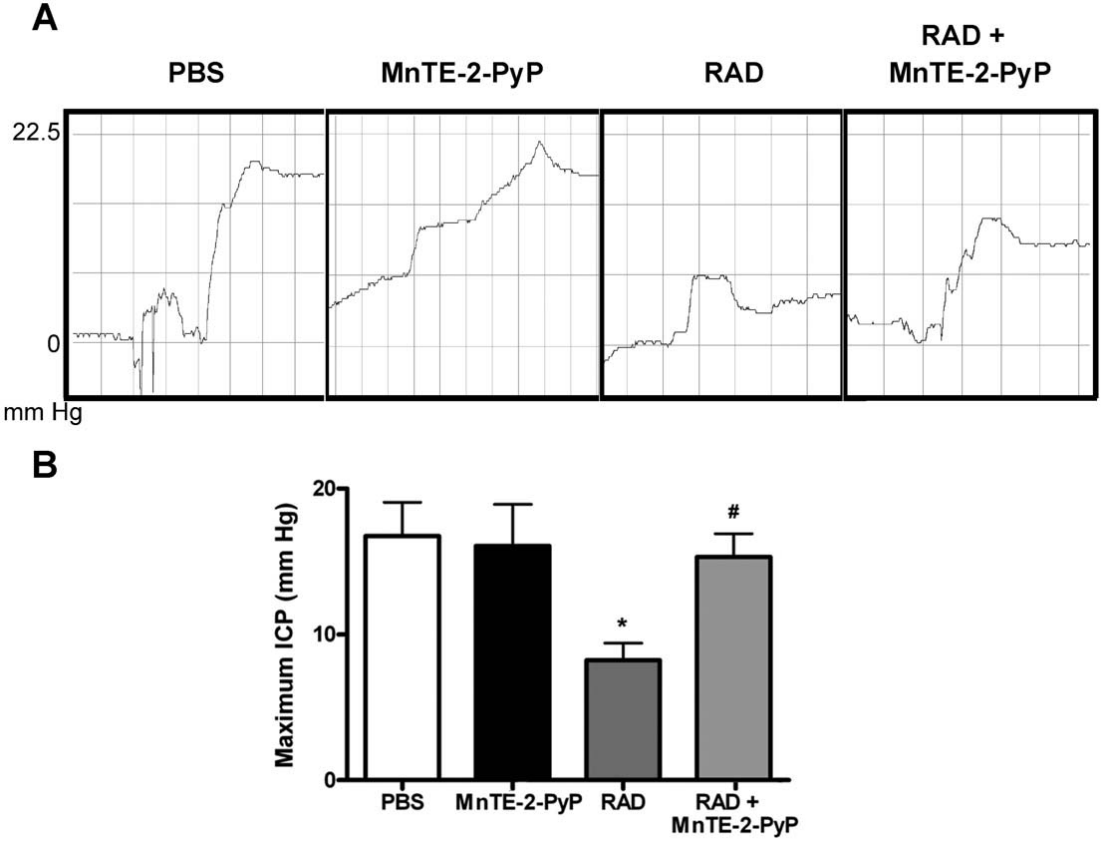
Measuring erectile function in rats. Intracavernous pressure (ICP) was obtained after cavernous nerve stimulation as a measurement of erectile function 12 weeks post-irradiation. A. Representative pressure curves obtained after nerve stimulation. B. Maximum ICP obtained after cavernous nerve stimulation. Irradiation caused a significant decrease in ICP (RAD group) as compared to the non-irradiated group (PBS). MnTE-2-PyP significantly protected from the irradiation-induced loss in ICP (MnTE-2-PyP RAD). n = 8 rats/group, asterisk (*) denotes significant difference from PBS group, p<0.05 and the number symbol (#) denotes significant difference from RAD group, p<0.05.

Erectile dysfunction is a common side-effect associated with prostate cancer radiation therapy. In our radiation model, we observed significant reduction in the intracavernous pressure in rat penises 12 weeks post-irradiation as compared to non-irradiated saline controls (Figure 7). A reduction in intracavernous pressure is a direct measure of erectile dysfunction. Irradiated rats lost >50% of their intracavernous pressure; however, the irradiated rats receiving MnTE-2-PyP were completely protected from intracavernous pressure loss. Thus, MnTE-2-PyP prevents radiation-induced developing erectile dysfunction.

**Figure 8:**
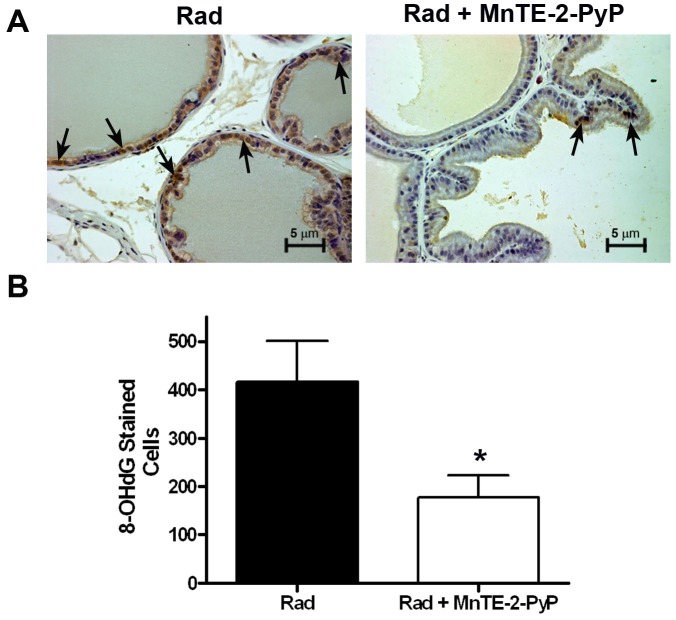
MnTE-2-PyP reduces DNA oxidative damage induced by irradiation in prostate epithelial cells. Irradiation is a known inducer of 8-OHdG, a marker of DNA oxidative damage. MnTE-2-PyP significantly reduced 8-OhdG in the prostate of irradiated animals 6 weeks post-irradiation. n = 4 rats/group, asterisk (*) denotes a significant difference from the RAD group.

Radiation is known to cause oxidative stress and 8-hydroxy-2-deoxyguanosine (8-OHdG) is a marker for DNA oxidative damage. Others have shown that radiation induces 8-OHdG in the nuclei of affected cells [7]. We measured 8-OHdG via immunostaining in the rat prostates 6 weeks post-irradiation. In accordance with previous studies, we observed numerous nuclear positively stained cells for 8-OHdG in the irradiated alone group. However, in the presence of MnTE-2-PyP a significant reduction in 8-OHdG staining was observed (Figure 8). Thus, MnTE-2-PyP may protect from radiation- induced damage in part by inhibiting DNA oxidative stress.

## Discussion

In this study, we have shown that MnTE-2-PyP given i.p. reaches the urogenital system and is present in concentrations high enough to protect the urogenital system from oxidative damage. We demonstrate that MnTE-2-PyP protects from many side-effects associated with prostate cancer radiation therapy. Specifically, MnTE-2-PyP reduces overall weight loss, skin damage and testicular atrophy associated with lower abdominal radiation exposure. Furthermore, MnTE-2-PyP protects the normal prostate and penile tissues from irradiation damage. These penile tissues had less fibrosis and the prostate tissues displayed less epithelial atrophy in irradiated rats treated with MnTE-2-PyP as compared to rats irradiated alone. Importantly, we also demonstrate that MnTE-2-PyP can prevent radiation-induced erectile dysfunction. Thus, MnTE-2-PyP protects a variety of tissues from irradiation damage and preserves the function of these organs as well.

One mechanism by which MnTE-2-PyP could be protecting the irradiated tissues from damage is by inhibiting ROS in bystander tissues. In our studies, MnTE-2-PyP significantly inhibited DNA oxidative damage via measurement of 8-OHdG in irradiated rats. Because MnTE-2-PyP is a known scavenger of superoxide and hydrogen peroxide, we speculate the MnTE-2-PyP is protecting the DNA in the bystander tissues by scavenging these reactive oxygen species, which ultimately prevent fibrosis of normal tissues.

In order for a drug to be a good radioprotectant in conjunction with cancer treatment, the drug cannot protect the tumor from radiation-induced death. Based on results obtained from previous studies, MnTE-2-PyP further reduces the growth of tumors in the presence of radiation [21]. Therefore, MnTE-2-PyP appears to synergize with radiation therapy to combat the prostate tumor and does not harm or diminish the effect of prostate radiation therapy.

**Figure 9:**
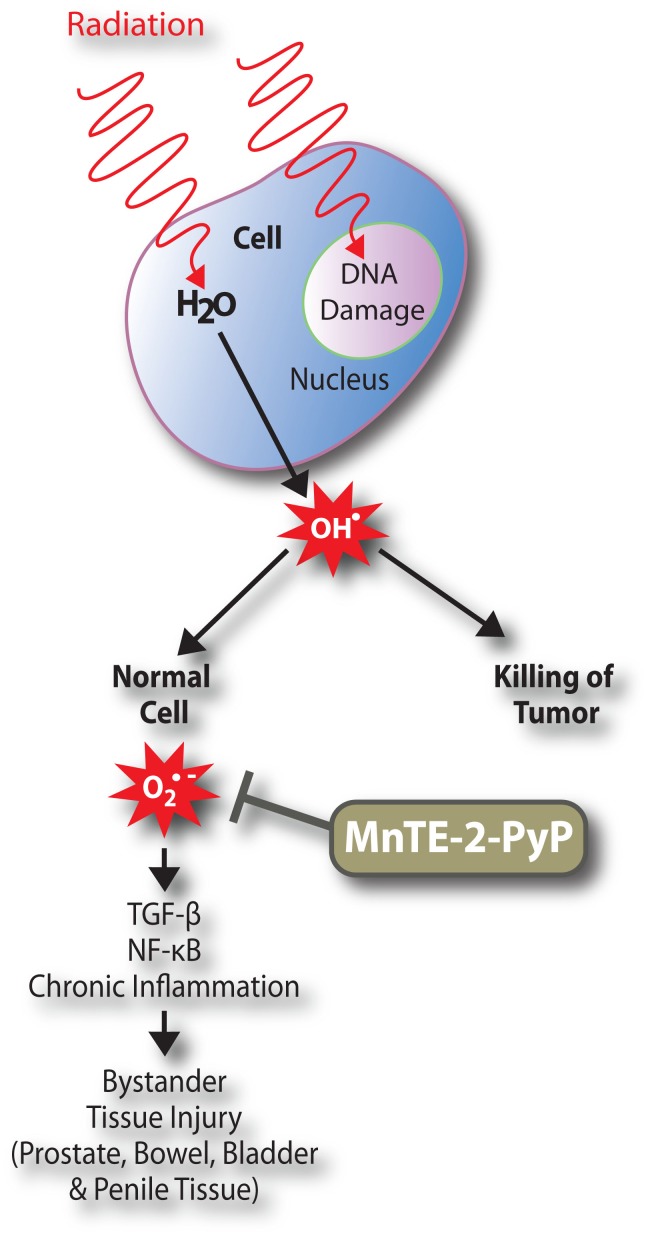
A model demonstrating how MnTE-2-PyP could work as a radioprotectant. We have shown that the potent antioxidant, MnTE-2-PyP protects normal tissues from irradiation-induced damage. We hypothesize that MnTE-2-PyP can inhibit injury of healthy bystander tissues by inhibiting inflammation driven by oxidative stress. We also hypothesize that MnTE-2-PyP has no effect on the killing of tumor cells due to the inability of MnTE-2-PyP to scavenge the damaging hydroxyl radical release caused by irradiation. Thus, MnTE-2-PyP can protect normal tissue from irradiation damage while not compromising the ability of irradiation to effectively kill tumor cells.

In Figure 9, we present a working hypothesis as to how MnTE-2-PyP both inhibits tumor growth but protects from normal tissue damage. The major component of any cell is H_2_O. Therefore, when ionizing radiation is aimed at a tumor it will likely interact with H_2_O and produce the highly reactive hydroxyl radical (OH^·^). The hydroxyl radical causes DNA damage, cell death, and growth inhibition of the tumor cells and the rate constant for the hydroxyl radical is extremely fast, 10^9^–10^10^ M^−1^s^−1^. MnTE-2-PyP is kinetically too slow (rate constant ∼10^7^ M^−1^s^−1^) to scavenge the hydroxyl radical, so the initial direct irradiation of the tumor will not be affected by MnTE-2-PyP, and due to tumor cell death, the tumor will shrink.

The direct effects of radiation are acute; however, the majority of side-effects caused by irradiation exposure occur more slowly over time. This type of secondary damage is termed the “by-stander effect” of irradiation damage, which is also shown to be driven by free radicals [24]. Superoxide (O_2_^·−^) and hydrogen peroxide (H_2_O_2_) are thought to be the main drivers in causing the more long term inflammation associated with bystander tissue damage. Superoxide and hydrogen peroxide are kinetically much slower acting than the hydroxyl radical; thus, MnTE-2-PyP can adequately scavenge both of these free radicals [10], [13]. MnTE-2-PyP is an anti-inflammatory agent as well. MnTE-2-PyP can inhibit NF-κB signaling in a variety of disease models [16], [27]. Gauter-Fleckenstein *et al.* recently showed in a lung radiation therapy model that MnTE-2-PyP inhibits inflammation by reducing the fibrogenic cytokine, TGF-β [26]. Thus, we postulate that MnTE-2-PyP is suppressing oxidative stress in the bystander tissues, which reduces key transcription factors that drive inflammation (Figure 9).

Radiation of prostatic tumors is a very successful method to treat prostate cancer. Since radiation works so well to kill the prostatic tumor, physicians may be leery of using therapeutics that protect normal tissues from irradiation damage with the fear that the tumor will be protected as well. However, the side-effects caused by irradiation of prostate tumors are a major concern to the patient. With increased early detection and treatment of localized prostate cancer, combined with increased patient longevity, the need to address chronic, long-term consequences of radiation treatment is increasing. The most problematic side-effects are declined bowel function when the pelvis requires irradiation (affecting 10% of men receiving prostatic irradiation), rectal wall injury and erectile dysfunction (affecting up to 63% of individuals receiving prostate irradiation therapy at two years post treatment) [4], [28]. Based on the data from this study and the combination with previous studies, we believe that the addition of MnTE-2-PyP with radiation therapy would adequately protect the surrounding normal tissue from damage in addition to enhancing the tumorcidal effects.

In conclusion, our data suggests that the antioxidant, MnTE-2-PyP, is a potent radioprotectant and in combination with prostate cancer irradiation will help to protect normal urogenital tissues from damage. We believe that this agent warrants serious attention for clinical development, not only for pelvic irradiation but for sites where radiation is commonly used to treat tumors: GI, thorax, head and neck areas. We believe this compound has the potential to dramatically improve the quality of life for patients undergoing radiation therapy for prostate cancer without sacrificing the effectiveness of radiation-induced killing of the tumor.
